# Continuous radiofrequency thermocoagulation under CT-guidance for glossopharyngeal neuralgia: Two case reports: Erratum

**DOI:** 10.1097/MD.0000000000011465

**Published:** 2018-07-06

In the article, “Continuous radiofrequency thermocoagulation under CT-guidance for glossopharyngeal neuralgia: Two case reports”,^[1]^ which appeared in Volume 97, Issue 24 of *Medicine*, the affiliation for corresponding author Dr. Tao Sun appeared incorrectly and should have been “Department of Pain Management, Shandong Provincial Hospital Affiliated to Shandong University, Jinan, Shandong, China.”
